# Virtual reality simulation training improve diagnostic knee arthroscopy and meniscectomy skills: a prospective transfer validity study

**DOI:** 10.1186/s40634-023-00688-8

**Published:** 2023-12-14

**Authors:** Alexandre Tronchot, Tiphaine Casy, Nicolas Vallee, Harold Common, Hervé Thomazeau, Pierre Jannin, Arnaud Huaulmé

**Affiliations:** 1 University Rennes, CHU Rennes, Inserm, LTSI, Equipe MediCIS- UMR 1099, 35000 Rennes, France; 2 Orthopaedics and Trauma Department, Rennes University Hospital, 2 Rue Henri Le Guilloux, 35000 Rennes, France

**Keywords:** Surgical training, Arthroscopy simulator, Virtual reality, Transfer validity

## Abstract

**Purpose:**

Limited data exist on the actual transfer of skills learned using a virtual reality (VR) simulator for arthroscopy training because studies mainly focused on VR performance improvement and not on transfer to real word (transfer validity). The purpose of this single-blinded, controlled trial was to objectively investigate transfer validity in the context of initial knee arthroscopy training.

**Methods:**

For this study, 36 junior resident orthopaedic surgeons (postgraduate year one and year two) without prior experience in arthroscopic surgery were enrolled to receive standard knee arthroscopy surgery training (NON-VR group) or standard training plus training on a hybrid virtual reality knee arthroscopy simulator (1 h/month) (VR group). At inclusion, all participants completed a questionnaire on their current arthroscopic technical skills. After 6 months of training, both groups performed three exercises that were evaluated independently by two blinded trainers: i) arthroscopic partial meniscectomy on a bench-top knee simulator; ii) supervised diagnostic knee arthroscopy on a cadaveric knee; and iii) supervised knee partial meniscectomy on a cadaveric knee. Training level was determined with the Arthroscopic Surgical Skill Evaluation Tool (ASSET) score.

**Results:**

Overall, performance (ASSET scores) was better in the VR group than NON-VR group (difference in the global scores: *p* < *0.001*, in bench-top meniscectomy scores: *p* = *0.03*, in diagnostic knee arthroscopy on a cadaveric knee scores: *p* = *0.04*, and in partial meniscectomy on a cadaveric knee scores: *p* = *0.02)*. Subgroup analysis by postgraduate year showed that the year-one NON-VR subgroup performed worse than the other subgroups, regardless of the exercise.

**Conclusion:**

This study showed the transferability of the technical skills acquired by novice residents on a hybrid virtual reality simulator to the bench-top and cadaveric models. Surgical skill acquired with a VR arthroscopy surgical simulator might safely improve arthroscopy competences in the operating room, also helping to standardise resident training and follow their progress.

**Level of evidence:**

2

**Supplementary Information:**

The online version contains supplementary material available at 10.1186/s40634-023-00688-8.

## Introduction

Due to the teachers and students’ time constraints, the traditional orthopaedic surgical training that relies on the mentor-apprentice model seems to have reached a limit. This is particularly true for high-demanding surgical techniques, such as arthroscopy [24] in which limited motion in a narrow joint space is combined with non-intuitive hand–eye coordination [26]. Surgical simulation allows surgeons to safely learn technical skills outside the operating room [11]. Computer-based simulation, such as hybrid virtual reality (VR) devices, is particularly suitable to learn arthroscopic technical skills and to obtain quantitative data for performance/skill evaluation and progress follow-up [2, 27, 33]. However, most of the literature is based on face and construct validity and only few data report the transferability of the skills learned with a VR simulator [19] to the real world (i.e., transfer validity). Moreover, studies mainly focused on performance/skill improvement [25, 33] directly assessed on the simulator itself with the risk of game-like performance. Several global rating scales for arthroscopic surgery have been developed in the last decade [4, 13, 16, 30]. The Arthroscopic Surgical Skill Evaluation Tool (ASSET) is widely used [14] to compare bench-top models [20], cadaver studies [16], VR simulators [21], and also during real surgical intervention [17]. Given that evaluating students directly on patients raises ethical and safety considerations,cadaveric models are considered the closest to a real intervention [15].

The purpose of this single-blinded, controlled trial was to objectively investigate transfer validity in the context of initial knee arthroscopy training. We hypothesized that junior orthopaedic surgery residents trained for knee arthroscopy (diagnosis and partial meniscectomy) with a hybrid VR simulator better transfer their surgical technical skills to bench-top models and cadavers than residents who follow the current national training program.

## Materials and methods

This study complies with the national reference methodology MR-004 for the use of personal data for research, was approved by the National Commission for Information Technology and Civil Liberties, and was registered in the national database. No external funding was received for this study.

### Study population

Between November 2020 and January 2021, thirty-six junior and novice orthopaedic surgery residents (postgraduate year one and two, PGY-1 and PGY-2) from five different hospitals of the local Orthopaedic Universitary Network participated in a theoretical and practical course on the basics of arthroscopy: a didactic lecture by a senior surgeon specialized in arthroscopy followed by a practical course (30 min per participant) using a hybrid VR simulator with the same instructor for all. Then, they were all enrolled in a 6-month study during which they received either the standard national training program [3] on knee arthroscopy surgery (NON-VR group) or this standard training plus six additional training sessions (1 h per month) on a hybrid VR knee arthroscopic simulator (VR group). At inclusion, all participants completed a questionnaire on their arthroscopic technical skills and performed a diagnostic knee arthroscopy and a meniscectomy using the VR knee arthroscopy simulator to confirm their novice expertise level. Inclusion in both groups was not randomized because of geographical and travel time considerations, as initial VR training and final evaluation were done at one site only. Informed consent was obtained from all participants. The VR group included 16 residents (*n* = 8 PGY-1, *n* = 8 PGY-2) and the NON-VR group included 20 residents (*n* = 10 PGY-1, *n* = 10 PGY-2).

### Training protocol

**Fig. 1:**
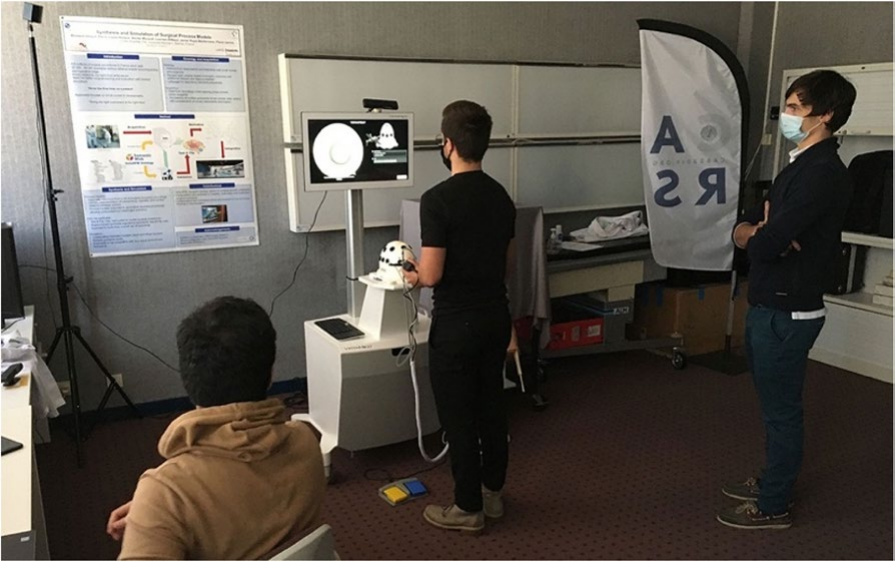
Training session for a trainee in the VR group

The VR group followed a fixed standardized training program with increasing difficulty using the VirtaMed AG (Schlieren) ArthroS™ arthroscopy simulator. This hybrid VR simulator with passive haptic (feeling of resistance, without robotic force feedback) has several modules that combine physical interface and computer software. The face and construct validity of the simulator and its different modules were assessed in previous studies [8, 28, 31]. The study authors organized and funded the travel of residents in groups of 3 at most to follow the supervised VR training (1 h per month) in the same designated laboratory with the same instructor for 6 months (Fig. 1). This training became increasingly more difficult with the aim of acquiring the basics of knee arthroscopy, such as *triangulation, periscoping, centring and camera alignment, bimanual dexterity, diagnostic arthroscopy of the knee* (the complete program is available in Additional file 1). The NON-VR group followed the national standard training only, with various theoretical and occasional practical courses in arthroscopy [3]. Thus, they were asked not to practice on a VR arthroscopy simulator during the whole study period.

### Global rating scale

Arthroscopy training was assessed using the ASSET score. This tool was chosen because it has been used in many previous studies [5–7, 17]. The scale was translated with the help of two independent evaluators, experts in knee arthroscopic surgery and teaching (Additional file 2). The original checklists for the diagnostic knee arthroscopy and meniscectomy were modified to make the procedure as standard and reproducible as possible. Before the final evaluation of the 36 residents, both evaluators practiced scoring twice (at an interval of two weeks) by assessing videos of diagnostic knee arthroscopy and meniscectomy on the hybrid VR simulator by ten anonymous participants, not included in the study population.

### Evaluation

**Fig. 2:**
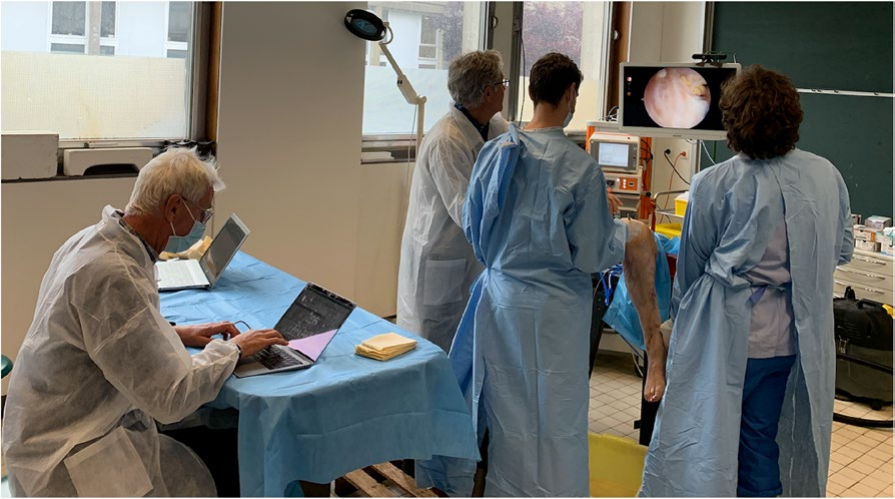
Evaluation session with both evaluators, the trainee and a surgical assistant

After 6 months of training, both groups performed three exercises: i) an arthroscopic partial meniscectomy on a bench-top knee simulator; ii) a supervised diagnostic knee arthroscopy on a cadaveric knee; and iii) a supervised partial meniscectomy cadaveric knee. Their performance was assessed independently by both blinded evaluators (not involved in their standard and VR-based training) using the ASSET score (Fig. 2). Each participant completed a second questionnaire on their newly acquired expertise in arthroscopy in the past 6 months and their feeling of progress in arthroscopy. Each participant was given instructions and a video demonstration of a diagnostic knee arthroscopy one week before the evaluation. An arthroscopy knee bench-top simulator (Arthroscopy Dry Knee, Sawbones, Malmö, Sweden) and 36 disposable menisci (Menisci Insert, Normal Anatomy, Off-White 35a Elastomer, Sawbones, Malmö, Sweden) were set up in one room of the cadaver laboratory at the institute. Another room was dedicated to the evaluation using 20 cadaveric knees (approximately one knee for two evaluations, depending on the preservation quality; all cadavers were from donations to the university anatomy program). Portal landmarks and incisions were made by both assessors, and they performed a first assessment to establish the same notation for the "Added complexity to the procedure" item in the ASSET score. In both rooms, a standard 30 ° arthroscope with an arthroscopic camera and display system (Smith & Nephew Endoscopy, Huntingdon, United Kingdom) was used for all participants.

### Statistical analysis

Sample size was computed for the null hypothesis “performance is equal in the VR group and NON-VR group” with acceptable type 1 and type 2 errors, $\alpha$= *0.05* and $\beta$ = *0.20* respectively, *power* = *0.8*. For this calculation, the ASSET scores from a previous arthroscopy validation study were used [16]. This gave a minimum number of 13 participants for each group, in line with previous transfer validation studies [11, 27]. Descriptive statistics were used to describe demographic data: mean,median, standard deviation and interquartile range for continuous variables (i.e., age, simulator score, ASSET score) and percentages for categorical variables (i.e., sex, dominant side, year of residency, arthroscopic experience, arthroscopic training, simulator training). Group differences for discrete variables were evaluated using the Chi-square or Fisher’s exact test. The data distribution was assessed with the Kolmogorov–Smirnov test; then, normally distributed data were compared with the parametric *t*-test. The non-parametric Mann–Whitney test was used in the other cases. For all comparisons, a *p* value < 0.05 was considered significant. The PGY-1 and PGY-2 subgroups were also compared. All statistical analyses were done with Excel (version 16.32, Microsoft, Seattle, United States).

## Results

### Study groups

Age, residency year, and diagnostic knee arthroscopy/meniscectomy performance level using the VR simulator and also arthroscopic experience at inclusion were comparable in the VR and NON-VR groups (Table 1). At inclusion, all participants had performed less than five arthroscopic surgeries as principal operator, often under the supervision of a senior surgeon. Most of them never practiced (77.8%) on real patient. Overall, the sample included more men than women, but the sex ratio was comparable between groups. Comparison of the arthroscopy experience at the end of the 6-month training period (Table 2) showed that participation in arthroscopy surgeries as an assistant was comparable between groups, but the percentage of participants who had already performed arthroscopic surgery as principal operator tended to be higher, although not significant and still inferior to 5 surgeries, in the VR group. Feeling of progress in arthroscopic skills was significantly higher in the VR group.

**Table 1:** Participants’ characteristics at inclusion

	**Non-VR Group (*n* = 20)**	**VR Group (*n* = 16)**	**Total (*n* = 36)**	***P*-Value**
Age, years	25.3 [24–27]	25.4 [24–29]	25.3 [24–29]	0.77^*^
Sex				0.48^†^
*Men*	13 (65%)	13 (81.2%)	26 (72.2%)	
*Women*	7 (35%)	3 (18.8%)	10 (27.8%)	
Dominant side				1^†^
*Right*	17 (85%)	14 (87.5%)	31 (86.1%)	
*Left*	3 (15%)	2 (12.5%)	5 (13.9%)	
*Ambidextrous*	0 (0%)	0 (0%)	0 (0%)	
Year of residency				1^†^
*PGY-1*	10 (50%)	8 (50%)	18 (50%)	
*PGY-2*	10 (50%)	8 (50%)	18 (50%)	
Arthroscopic experience before inclusion				
Surgical assistant (< 100 arthroscopies)				0.91^†^
*Yes*	20 (100%)	15 (93.8%)	35 (97.2%)	
*No (ie. never assisted arthroscopic surgery)*	0 (0%)	1 (6.2%)	1 (2.8%)	
Principal operator (< 5 arthroscopies)				0.96^†^
*Yes*	5 (25%)	3 (18.8%)	8 (22.2%)	
*No (ie. never practiced arthroscopy)*	15 (75%)	13 (81.2%)	28 (77.8%)	
Arthroscopic training before inclusion				0.95^†^
*Yes (Cadaveric Model)*	3 (15%)	3 (18.8%)	6 (16.7%)	
*Yes (1 h, Bench-top Model)*	3 (15%)	2 (13.2%)	5 (13.9%)	
*No*	14 (70%)	11 (68.8%)	25(69.4%)	
VR training before inclusion				0.94^†^
*Yes*	6 (30%)	5 (31.2%)	11 (30.6%)	
*No*	14 (70%)	11 (68.8%)	25 (69.4%)	
VR arthroscopic simulator score at inclusion	125 [116–134]	123 [111–135]	124 [113–135]	0.14^*^

Data are reported as median [interquartile range] or numbers (percentage)

^*^Independent-samples t-test

^†^Pearson’s chi-square test

**Table 2:** Participants’ characteristics at the study (evaluation time)

	**Non-VR Group (*n* = 20)**	**VR Group (*n* = 16)**	**Total (*n* = 36)**	***P*-Value**
Arthroscopic experience during the 6 months				
Surgical Assistant (< 100 arthroscopies)				1^†^
*Yes*	18 (90%)	14 (87.5%)	32 (88.9%)	
*No (ie. never assisted arthroscopic surgery)*	2 (10%)	2 (12.5%)	4 (11.1%)	
Principal operator (< 5 arthroscopies)				0.23^†^
*Yes*	5 (25%)	8 (50%)	13 (36.1%)	
*No (ie. never practiced arthroscopic surgery)*	15 (75%)	8 (50%)	23 (63.9%)	
ARTHROSCOPIC training during the 6 months				0.48^†^
*Yes (Cadaveric Model)*	0 (0%)	0 (0%)	0 (0%)	
*Yes (1 h, Bench-Top Model)*	4 (20%)	1 (6,2%)	5 (13.9%)	
*No*	16 (80%)	15 (93.8%)	31 (86.1%)	
Feeling of progress in arthroscopic skills				< 0.001^†^
*Yes*	7 (35%)	16 (100%)	23 (63.9%)	
*No*	13 (65%)	0 (0%)	13 (36.1%)	

Data are reported as numbers (percentage)

^†^Pearson’s chi-square test

### ASSET

**Fig. 3:**
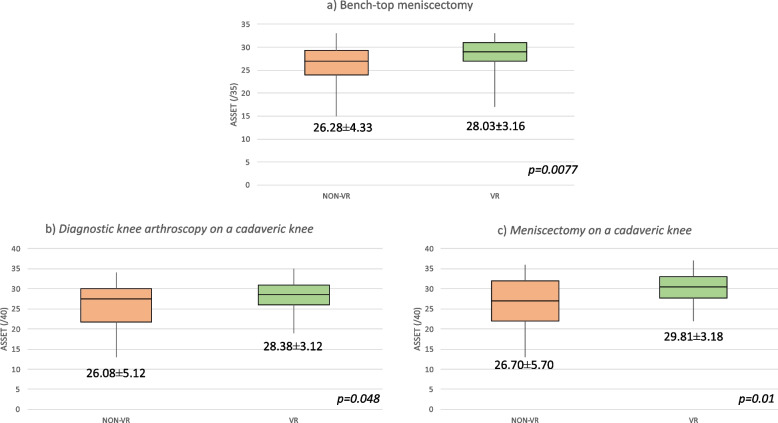
Box-plots showing the ASSET score for each exercise in the VR and NON-VR groups. **a** Bench-top meniscectomy;** b** Diagnostic knee arthroscopy on a cadaveric knee; **c** Meniscectomy on a cadaveric knee. Data are reported as median [interquartile range]

The 4 ASSET scores (i.e., global, bench-top meniscectomy, diagnostic arthroscopy and meniscectomy on cadaveric knee scores) were significantly higher in the VR than NON-VR group (mean ± standard deviation]): 28.74 ± 3.75 versus 26.37 ± 5.55 (*p* = *0.002*), 28.03 ± 3.16 *versus* 26.28 ± 4.33 (*p* = *0.077*), 28.38 ± 3.12 *versus* 26.08 ± 5.12, (*p* = *0.048*), and 29.81 ± 3.18 *versus* 26.70 ± 5.70, (*p* = *0.01*) (Fig. 3)*.* The ASSET score internal consistency was good for both evaluators (Cronbach’s alpha: *0.90*), but the inter-observer reproducibility was moderate (intra-class coefficient correlation: *0.66* for the global score, *0.68* for the bench-top meniscectomy score, *0.61* for the diagnostic knee arthroscopy on cadaveric knee score, and *0.71* for the meniscectomy on cadaveric knee score).

### Subgroup analysis

**Fig. 4:**
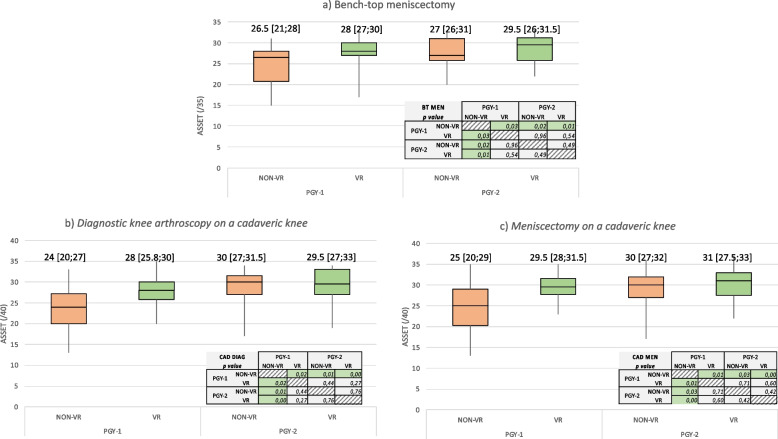
Box plots showing the subgroup analysis results (ASSET scores for PGY-1 and PG-2 participants in the VR and NON-VR groups). **a** Bench-top meniscectomy; **b** Diagnostic knee arthroscopy on a cadaveric knee; **c** Meniscectomy on a cadaveric knee. Data are reported as median [interquartile range]. BT MEN, bench-top meniscectomy; CAD DIAG, knee diagnostic arthroscopy on cadaveric knee; CAD MEN, meniscectomy on cadaveric knee

For the subgroup analysis, participants in the two groups were divided in function of their postgraduate year. All four ASSET scores of the VR PGY-1 subgroup were significantly higher than in the NON-VR PGY-1 subgroup. Conversely, scores were not different between the VR PGY-1 and the PGY-2 subgroups (VR and NON-VR). (Fig. 4).

## Discussion

The most important finding of the present study was that our results confirmed the transfer validity of the skills acquired during hybrid VR simulator training to cadaveric model for diagnostic knee arthroscopy and partial meniscectomy. These results reinforce the findings of previous studies. Specifically, Howells et al. [11] confirmed the transfer validity of bench-top models (sustained one-week arthroscopic training program) to the operating room but only for diagnostic knee arthroscopy. Rebolledo et al. [27] found significant improvement in diagnostic shoulder arthroscopy, but only a non-significant trend for diagnostic knee arthroscopy in participants who underwent a short two and half-hour arthroscopic training using another VR simulator. The face and construct validity [8, 25, 31] of VR simulators have already been proven even if they still have controversies [32]. Validating the transfer of skills acquired with this type of simulator confirmed the contribution of these tools to the initial training of orthopaedic surgeons. In a descriptive survey on the arthroscopy training and acquired skills, Pioger et al. [22] found that 40% of year-four orthopaedic surgery residents had performed less than five simple arthroscopies as main operator. Moreover in that specific national training program, ~ 69% thought that they would not have acquired enough skills by the end of their registrar training. Our findings showed that the training on a VR simulator allowed gaining skills and also confidence. In a recent study [29], residents from different European countries were surveyed and even wanted an approximate average mandatory training time of 42 h per year. It is currently difficult to determine the learning curve of a simple procedure such as arthroscopic knee exploration and meniscectomy learned on a simulator. Some studies conclude that 2.5 h are sufficient to teach medical students [2] while other studies estimate that 10 h of training on a simulator are insufficient for novice residents to reach the level of experienced surgeons [1]. Our training program (one hour per month for 6 months) led to a significant improvement of both diagnostic knee arthroscopy and meniscectomy. This study shows the positive impact of this patient-safe practical training in arthroscopy on a VR simulator that can be easily integrated into the curriculum of novice orthopaedic surgery residents, leaving room for the other training modules (traumatology, arthroplasty, microsurgery, etc.…).

### Limitations

First, although there was no randomization due to geographical constraints, we limited this bias by creating comparable groups with similar experience levels at inclusion. We included all first- and second-year orthopaedic surgery residents from our regional network because arthroscopy training is now part of the national curriculum. This allowed equally distributing residents who were interested or not in arthroscopy in the two groups, thus limiting the selection bias that could lead to the inclusion mostly of participants who are interested in arthroscopy and/or VR training. Second limitation, we found moderate inter-operator reliability. However, previous studies [2, 11] assessed the trainee’s performance at the training end by one evaluator only. Rebolledo et al. [27] did not analyze the inter-operator reliability of the two raters. We decided to choose two evaluators, experts in knee arthroscopy but with a different trainer profile: one from a university hospital (i.e. used to supervise residents with different expertise levels, interested or not in arthroscopy) and one from a clinic specialized in sports surgery (i.e. used to supervise fellows, more advanced in their studies and most often very interested in arthroscopy). During the evaluation process, both assessors did not share their scores except for the procedure difficulty. Third, some studies [6, 23] have shown that skills are not or very little maintained over time after technical training on a simulator, questioning the interest of short condensed training courses, as usually proposed in studies (one day training during a convention [12], or one week training during a study [18]). Therefore, we chose a longer training program (1 h per month) to address this issue. We found that this allowed improving skills as indicated by the ASSET scores at the end of the program, particularly compared with the NON-VR group. On the other hand, results were not conclusive for PGY-2 residents. Fourth limitation of our study is the absence of follow-up after the study end. Indeed, it would be important to re-assess participants after one year to determine whether differences remain between groups/subgroups, and whether the VR group still perform better at more complex procedures [9]. Fifth, subgroup analysis showed that this training was most beneficial for the VR PGY-1 subgroup that reached the level of the PGY-2 subgroups. This VR simulator program may be sufficient for novices, but may require improvements (e.g. introduction of more complex procedures) for more experienced trainees [10] or more training hours as suggested by Anetzberger et al. where 10 h of training was not enough for their study [1]. Finally, the ASSET scores were, on average, 10 points higher than the expected results for participants with that level of expertise in a previous study [16]. This difference could be due to the fact that in the study by Koehler et al. [16] scoring was done using arthroscopy videos and not live. A hypothesis is that the fact that the evaluator saw the evaluated resident in person might bias the result by upgrading the rating compared with a video evaluation.

## Conclusion

This study showed the transferability of the skills acquired by novice residents on a hybrid virtual reality simulator to the bench-top and cadaveric models. Surgical skill acquired with a VR arthroscopy surgical simulator might safely improve arthroscopy competences in the operating room, also helping to standardise resident training and follow their progress.

### Supplementary Information

**Additional file 1. **VR training protocol.

**Additional file 2. **ASSET Score.
