# Supplementary material for: Virtual reality simulation training improve diagnostic knee arthroscopy and meniscectomy skills: a prospective transfer validity study
Source: J Exp Orthop. 2023 Dec 14;10:138. doi: 10.1186/s40634-023-00688-8 (PMC10721743; doi:10.1186/s40634-023-00688-8)
Supplement: Supplementary file 1 — Additional file 1. VR training protocol. [file 40634_2023_688_MOESM1_ESM.docx]

**VR TRAINING PROTOCOL**

- **TRAINING SESSION 1 (**common for both VR and NON-VR groups)***
- FAST MODULE
  - *Horizon*
  - *Centering*
  - *Telescoping*
  - *Periscoping*
  - *Image centering and Periscoping*
  - *Image Probing* (wide field of view)
  - *Remove the stars*
- (*RIGHT) KNEE MODULE
  - *Guided Diagnostic arthroscopy II*
  - *Guided Meniscectomy II* (x3)
- **TRAINING SESSION 2**
- FAST MODULE
  - *Centering*
  - *Telescoping*
  - *Tracing the lines*
  - *Periscoping*
  - *Image centering and Periscoping*
  - *Image Probing,* *wide field of view*
  - *Removing the stars*
- KNEE MODULE
  - ***Diagnostic Tour II***
  - *Diagnostic Tour II & Palpation*
  - *Hook all rings*
  - *Catch the stars*

- **TRAINING SESSION 3**
- FAST MODULE
  - *Telescoping*
  - *Traces the lines*
  - *Image centering and Periscoping*
  - *Collect the stars*
- KNEE MODULE
  - *AANA Guided Diagnostic*
  - *Diagnostic Tour II & Palpation (*without guidance arrow)*
  - *Catch the stars I*
  - *Diagnostic X (palpation of a peripheral medial meniscal lesion, answer to a questionnaire) (*without view help)*
  - *Synovitis I (with shaver)*
- **TRAINING SESSION 4**
- FAST MODULE
  - *Image centering and Periscoping* (arthroscope in **left hand)**
  - *Image centering (*arthroscope in **right hand)**
  - *Image centering and Periscoping* *(*arthroscope in **right hand)**
  - *Image Probing,* *wide field of view* *(*arthroscope in **right hand)**
- KNEE MODULE
  - *Diagnostic Tour II*
- SHOULDER MODULE
  - *Guided Diagnostic Tour Glenohumeral*
  - *Guided Diagnostic Tour Subacromial*
  - *Diagnostic Tour II & Palpation*
  - *Catch the stars I*
- **TRAINING SESSION 5**
- FAST
  - *Image centering and Periscoping* (arthroscope in **left hand)**
  - *Remove the stars* (arthroscope in **left hand)**
  - *Image centering and Periscoping* *(*arthroscope in **right hand)**
  - *Remove the stars (*arthroscope in **right hand)**
- KNEE 25’
  - *AANA Guided Diagnostic & Palpation*
  - *Guided Meniscectomy II*
  - *Diagnostic XVII (diagnostic tour and palpation of a ramp lesion by posteromedial approach, answer to a questionnaire) (*without view help)*
  - *Medial Meniscectomy with arthroscope in right hand (diagnostic tour then medial partial meniscectomy)*
  - *Bonus Exercise (Diagnostic Tour with 5 intra-articular loose body removal)*
