# Supplementary material for: Virtual reality simulation training improve diagnostic knee arthroscopy and meniscectomy skills: a prospective transfer validity study
Source: J Exp Orthop. 2023 Dec 14;10:138. doi: 10.1186/s40634-023-00688-8 (PMC10721743; doi:10.1186/s40634-023-00688-8)
Supplement: Supplementary file 2 — Additional file 2. ASSET Score. [file 40634_2023_688_MOESM2_ESM.docx]

| **Formulaire d'Évaluation Globale des Compétences en Arthroscopie ASSET-FR**  1  ***(Arthroscopic Surgical Skill Evaluation Tool)*** | |
| --- | --- |
| **1 - DEROULE SPECIFIQUE DES PROCEDURES EVALUEES** |  |

**1A** – MENISCECTOMIE SUR MAQUETTE SAWBONE

| **Résection méniscale standardisée / Méniscectomie partielle postérieure** | **Complété ?** | |
| --- | --- | --- |
| Mesure au crochet 1 cm de ménisque (*pour information, le crochet palpeur mesure 5mm*) | - Oui | - Non |
| Montre les limites du segment postérieur du ménisque | - Oui | - Non |
| Teste la stabilité de tout le ménisque du segment postérieur au segment antérieur | - Oui | - Non |
| Dessine au crochet les limites de la méniscectomie d'1 cm de longueur et d'une demi-largeur en profondeur que tu vas réaliser | - Oui | - Non |
| Réalise cette méniscectomie à la pince emporte-pièce (pince « basket ») | - Oui | - Non |
| Régularise les berges de la méniscectomie | - Oui | - Non |

**1B**- ARTHROSCOPIE DIAGNOSTIQUE SUR GENOU ANATOMIQUE

| **Arthroscopie diagnostique du genou** | **Complété ?** | |
| --- | --- | --- |
| Inspecter le cul-de-sac sous-quadricipital | - Oui | - Non |
| Inspecter et palper la patella (médial/latéral ; distal/proximal) | - Oui | - Non |
| Inspecter et palper la gorge trochléenne | - Oui | - Non |
| Inspecter la gouttière latérale | - Oui | - Non |
| Inspecter le tendon poplité et le récessus poplité | - Oui | - Non |
| Inspecter la gouttière médiale en repassant par le cul-de-sac sous-quadricipital | - Oui | - Non |
| Inspecter et palper le condyle fémoral médial | - Oui | - Non |
| Inspecter et palper le plateau tibial médial | - Oui | - Non |
| Inspecter et palper le ménisque médial (segments antérieur, moyen et postérieur) | - Oui | - Non |
| Inspecter et palper le Ligament Croisé Antérieur (LCA) / le Ligament Croisé Postérieur (LCP) | - Oui | - Non |
| Inspecter et palper le condyle fémoral latéral | - Oui | - Non |
| Inspecter et palper le plateau tibial latéral | - Oui | - Non |
| Inspecter et palper le ménisque latéral (segments antérieur, moyen et postérieur) | - Oui | - Non |

**1C**- MENISCECTOMIE SUR GENOU ANATOMIQUE

| **Résection méniscale standardisée / Méniscectomie partielle** | **Complété ?** | |
| --- | --- | --- |
| Mesure au crochet 1 cm de ménisque (*pour information, le crochet palpeur mesure 5mm*) | - Oui | - Non |
| Montre les limites du segment postérieur du ménisque | - Oui | - Non |
| Teste la stabilité de tout le ménisque du segment postérieur au segment antérieur | - Oui | - Non |
| Dessine au crochet les limites de la méniscectomie d'1 cm de longueur et d'une demi-largeur en profondeur que tu vas réaliser | - Oui | - Non |
| Réalise cette méniscectomie à la pince emporte-pièce (pince « basket ») | - Oui | - Non |
| Régularise les berges de la méniscectomie | - Oui | - Non |

| **Formulaire d'Évaluation Globale des Compétences en Arthroscopie ASSET-FR**  ***(Arthroscopic Surgical Skill Evaluation Tool)*** | | |
| --- | --- | --- |
| **ÉCHELLE D'EVALUATION GLOBALE**  **ARTHROSCOPIE DIAGNOSTIQUE** | | **Date de l’évaluation** :  … / … / … |
| **Votre Numéro d’Examinateur** : | **Identifiant du participant** : | **Numéro de la procédure** : |

Instructions : Pour chaque domaine de compétence, ENTOURER UN ÉNONCÉ dans chaque ligne décrivant le mieux dans quelle mesure l’opérateur a exécuté chaque aspect de cette procédure.

|  | ***1 – Débutant*** | ***2*** | ***3 - Compétent*** | ***4*** | ***5 - Expert*** |
| --- | --- | --- | --- | --- | --- |
| **Sécurité** | Dégâts importants du cartilage ou des tissus mous |  | Dégâts mineurs du cartilage ou des tissus mous |  | Pas de dégât du cartilage ou des tissus mous |
| **Champ de Vision** | Champ de vision étroit, positionnement inadéquat de l’arthroscope et/ou du foroblique |  | Champ de vision acceptable, positionnement adéquat de l’arthroscope et/ou du foroblique |  | Champ de vision étendu, positionnement optimal de l’arthroscope et/ou du foroblique |
| **Dextérité de la caméra** | Mouvements maladroits ou sans grâce, ne parvient pas à garder la caméra centrée et correctement orientée |  | Utilisation appropriée de la caméra, doit parfois se repositionner |  | Gracieux et habile tout au long de la procédure, avec une caméra toujours centrée et correctement orientée |
| **Dextérité instrumentale** | Maladroit avec les instruments, incapable d'orienter systématiquement les instruments vers la cible |  | Utilisation prudente et contrôlée des instruments, manque occasionnellement la cible |  | Utilisation sûre et précise de tous les instruments |
| **Coordination bimanuelle** | Incapacité à coordonner ses deux mains ou absence de coordination entre ses deux mains |  | Utilise ses deux mains mais échoue parfois à coordonner les mouvements de la caméra et des instruments |  | Utilise les deux mains pour coordonner le positionnement de la caméra et des instruments afin d'obtenir des performances optimales |
| **Fluidité de la procédure** | Arrêts fréquents, persiste sans progrès, nombreuses tentatives infructueuses avant d’achever les tâches |  | Progression régulière de la procédure opératoire avec peu de tentatives infructueuses avant d’achever les tâches |  | Déroulement évident de la procédure, transition fluide d'une tâche à l'autre sans aucune tentative infructueuse |
| **Qualité de la procédure** | Résultat final inadéquat ou incomplet |  | Résultat final adéquat, défauts mineurs qui ne nécessitent pas de correction |  | Résultat final optimal et sans défaut |
|  | ***1*** | | ***2*** | | ***3*** |
| **Complexité ajoutée à la procédure** | Aucune difficulté | | Difficulté modérée (légèrement inflammatoire et/ou tissus cicatriciels) | | Difficulté extrême (inflammatoire et/ou tissus cicatriciels importants, anatomie non conventionnelle) |
| **Autonomie** | Impossible de terminer la procédure même avec une ou plusieurs assistances | | Capable de mener à bien la procédure mais nécessitant une ou plusieurs assistances | | Capable de mener à bien la procédure sans assistance |

Selon vous, quel était le niveau de compétence de l’interne pour exécuter cette procédure ? (*Faire une marque verticale sur la ligne pour indiquer la compétence*)

Extrêmement compétent

Aucune compétence
